# A research pathway for the study of the delivery and disposition of nebulised antibiotics: an incremental approach from in vitro to large animal models

**DOI:** 10.1186/s40635-018-0180-7

**Published:** 2018-07-11

**Authors:** Jayesh A. Dhanani, Jeremy Cohen, Suzanne L. Parker, Hak-Kim Chan, Patricia Tang, Benjamin J. Ahern, Adeel Khan, Manoj Bhatt, Steven Goodman, Sara Diab, Jivesh Chaudhary, Jeffrey Lipman, Steven C. Wallis, Adrian Barnett, Michelle Chew, John F. Fraser, Jason A. Roberts

**Affiliations:** 1 Faculty of Medicine, UQ Centre for Clinical Research, The University of Queensland, Brisbane, Australia; 2 Department of Intensive Care Medicine, Royal Brisbane and Women’s Hospital, Brisbane, Australia; 3 Critical Care Research Group, The University of Queensland, Brisbane, Australia; 4 Advanced Drug Delivery Group, Faculty of Pharmacy, The University of Sydney, Sydney, New South Wales Australia; 5 Faculty of Science, School of Veterinary Science, The University of Queensland, Gatton, Australia; 6 Department of Nuclear Medicine and Specialised PET Services Queensland, Royal Brisbane and Women’s Hospital, Herston, Queensland Australia; 7 School of Medicine, Faculty of Health Sciences, University of Queensland, St Lucia, Queensland Australia; 8 Faculty of Health, Queensland University of Technology, Brisbane, Australia; 9 Institute of Health and Biomedical Innovation and School of Public Health and Social Work, Queensland University of Technology, Kelvin Grove, Brisbane, Australia; 10 Department of Anaesthesiology and Intensive Care, Department of Medical and Health Sciences, Linköping University, Linköping, Sweden; 11 Centre for Translational Anti-infective Pharmacodynamics, School of Pharmacy, The University of Queensland, Brisbane, Australia; 12 Department of Pharmacy, Royal Brisbane and Women’s Hospital, Brisbane, Australia

**Keywords:** Ventilator-associated pneumonia, Nebulised antibiotics, Inhaled mass, Particle size distribution, Regional drug distribution, Microdialysis

## Abstract

**Background:**

Nebulised antibiotics are frequently used for the prevention or treatment of ventilator-associated pneumonia. Many factors may influence pulmonary drug concentrations with inaccurate dosing schedules potentially leading to therapeutic failure and/or the emergence of antibiotic resistance. We describe a research pathway for studying the pharmacokinetics of a nebulised antibiotic during mechanical ventilation using in vitro methods and ovine models, using tobramycin as the study antibiotic.

**Methods:**

In vitro studies using a laser diffractometer and a bacterial-viral filter were used to measure the effect of the type and size of tracheal tubes and antibiotic concentration on the particle size distribution of the tobramycin 400 mg (4 ml; 100 mg/ml) and 160 mg (4 ml, 40 mg/ml) aerosol and nebulised mass delivered. To compare the regional drug distribution in the lung of two routes (intravenous and nebulised) of drug administration of tobramycin 400 mg, technetium-99m-labelled tobramycin 400 mg with planar nuclear medicine imaging was used in a mechanically ventilated ovine model. To measure tobramycin concentrations by intravenous and nebulised tobramycin 400 mg (4 ml, 100 mg/ml) administration in the lung interstitial space (ISF) fluid and blood of mechanically ventilated sheep, the microdialysis technique was used over an 8-h duration.

**Results:**

Tobramycin 100 mg/ml achieved a higher lung dose (121.3 mg) compared to 40 mg/ml (41.3 mg) solution. The imaging study with labelled tobramycin indicated that nebulised tobramycin distributed more extensively into each lung zone of the mechanically ventilated sheep than intravenous administration. A higher lung ISF peak concentration of tobramycin was observed with nebulised tobramycin (40.8 mg/l) compared to intravenous route (19.0 mg/l).

**Conclusions:**

The research methods appear promising to describe lung pharmacokinetics for formulations intended for nebulisation during mechanical ventilation. These methods need further validation in an experimental pneumonia model to be able to contribute toward optimising dosing regimens to inform clinical trials and/or clinical use.

## Background

Ventilator-associated pneumonia (VAP) is common in intensive care patients and is associated with high morbidity and mortality rates [1]. To improve outcomes, nebulised antibiotic therapy has been recommended for prevention [2] and treatment of VAP [3]. However, there is controversy, with recent guidelines not recommending the use of nebulised antibiotics, citing a lack of robust effectiveness data, possibly due to inadequate trial designs and/or ineffective drug delivery [4]. Antibiotic nebulisation can potentially be a viable alternative to conventional therapy for lung infection because if administered appropriately, it should enable high lung antibiotic concentrations without significant side effects [5]. However, there are a number of factors involved in achieving optimal delivery [6, 7], which need to be characterised to ensure desired concentrations are achieved. These include physicochemical properties of the antibiotics, tracheal tube size and type [7–9], besides factors associated with mechanical ventilation such as ventilator settings, circuit-related factors, nebuliser factors and patient-related factors such as airway geometry and patency [7]. Due to methodological limitations, the pharmacokinetics (PK) of nebulised antibiotics during mechanical ventilation is not well understood.

For effective nebulisation therapy with mechanical ventilation, it is essential to optimise these factors. Vital information regarding drug deposition, metabolism, absorption, kinetic profile and tolerability should be considered when designing optimised dosing regimens [10]. The clinical environment provides limited opportunities to accurately study these numerous factors affecting nebulised drug delivery and lung concentration of antibiotics [10]. Hence, in vitro studies are essential to study these factors, in isolation and in combination [11]. Further to this, animal models can allow the manipulation of variables and study of their effects, which may not be feasible in human subjects [12]. Although small animals have been extensively used for these purposes [13, 14], when the study involves factors that may significantly affect drug delivery such as the airway and lung anatomy as well as circulatory parameters, large animals are preferable as they more accurately represent the human scenario [15, 16]. The pathophysiological changes that occur in respiratory diseases could influence the PK of nebulised antibiotics [7]. Sheep are considered an appropriate large animal model to study pulmonary PK associated with different devices and/or formulations [10] in healthy and in experimental pneumonia models [17, 18]. In this paper, we describe an approach to providing a detailed characterisation of pulmonary PK of nebulised drug delivery using a combination of in vitro and ovine studies.

The specific aims of this research are:

1. To calibrate the nebulised delivery system during mechanical ventilation.
2. To quantify and compare the regional pulmonary distribution when administered via intravenous and nebulised routes during mechanical ventilation in a healthy ovine model.
3. To calibrate the microdialysis system and its application to quantify and compare lung interstitial fluid (ISF) concentrations of tobramycin when administered via intravenous and nebulised routes during mechanical ventilation in a healthy ovine model.

## Methods

The program of research comprises of complementary studies investigating different aspects of nebulised antibiotic therapy with a view to addressing key questions (Table 1). Each individual sub-study is described in detail below.

**Table 1:** Overview of the research pathway with the study components aimed at characterising the pharmacokinetics of nebulised tobramycin

Research pathway				
Study	1. In vitro particle sizing and inhaled mass study	2. In vivo lung distribution study	3. In vitro microdialysis recovery study	4. In vivo lung microdialysis study
Aims	Describing and comparing the aerosol characteristics of two formulations of tobramycin	Comparing lung distribution of i.v. vs nebulised radiolabelled tobramycin 400 mg	Comparing relative recovery of vancomycin and tobramycin with microdialysis	Comparing ISF, ELF and blood concentrations of i.v. vs nebulised tobramycin 400 mg
Design	In vitro simulated adult mechanical ventilation using • Laser diffraction • Inhaled mass	Mechanically ventilated healthy ovine model	In vitro study using simulated in vivo conditions	Mechanically ventilated healthy ovine model
Materials and methods	• Size 9.0 mm I.D. ETT and tracheostomy tube • 4 ml tobramycin 100 mg/ml (400 mg) and 40 mg/ml (160 mg) • Vibrating mesh nebuliser just proximal to the Y- piece • Triplicate experiments	• Technetium-99m-labelled tobramycin 400 mg • i.v. administration (*n* = 1) • Nebulisation (*n* = 1) • CT scan to derive lung outline • Gamma camera scanning—dorsal, ventral, bilateral • Tissue and blood sampling	• Vancomycin 5 μg/ml • Tobramycin 5 μg/ml • In 50 ml FFP solution and constant stirrer • Perfusate flow rates 1, 1.5 and 2 μl/min • Sample collection every 20 min for 100 min (*n* = 5) • Triplicate experiments	• Bilateral thoracotomy approach for insertion of microdialysis catheters • i.v. tobramycin 400 mg (*n* = 1) • Nebulised tobramycin 400 mg (*n* = 1) • Bronchoalveolar lavage (1 and 6 h) • Intravascular microdialysis • Sample collection every 20 min for 8 h (*n* = 24)
Analysis	Particle size distribution parameters: • dv10 (μm) • dv50 (μm) • dv90 (μm) • FPF (%) Inhaled mass parameters: • Inhaled drug percentage (%) • Lung dose (mg)	• P/C ratio • Dorsal: ventral ratio • Right: left lung ratio • Upper: middle: lower lung zone • Quantitative analysis in the lung, liver, kidney, blood and urine specimens	• Relative recovery values for each of the flow rates	• ELF concentration and PK • ISF concentration and PK • Plasma concentration and PK

*dv*_*10*_ volume diameter under which 10% of the sample resides; *dv*_*50*_ volume median diameter; *dv*_*90*_ volume diameter under which 90% of the sample resides; *FPF* fine particle fraction (particle size 1 to 5 μm); *inhaled drug percent* percent quantity of drug in the inhaled mass filter at the end of the tracheal tube post-nebulisation; *lung dose* the product of FPF and inhaled drug mass (mg); *ELF* epithelial lining fluid derived from urea levels; *ISF* interstitial space fluid; *PK* pharmacokinetics

### Calibrating the nebulised delivery system during mechanical ventilation

Two experimental techniques were employed for this purpose.

#### Aerosol particle size distribution

##### Equipment

We used an adult-use mechanical ventilator and circuits (Puritan Bennett 840 ventilator, Covidien-Medtronic, Mansfield, MA) in conjunction with a disposable vibrating mesh nebuliser (VMN) (Aeroneb pro, Aerogen, Inc., Galway, Ireland) placed in the inspiratory limb of the circuit as per the recommendations [19]. Although there are a number of delivery devices available, VMN is recommended for nebulised antibiotic therapy in mechanically ventilated patients [19, 20]. We investigated the effects of different concentrations of tobramycin (Tobra-day, Phebra) 100 mg/ml and 40 mg/ml (Tobramycin PF, Pfizer, Australia) on the nebulisation characteristics. We also investigated the effects of different types of tracheal tubes (endotracheal tube vs tracheostomy tube; Portex, Smiths Medical International Ltd., UK) with tube size 9.0 mm internal diameter.

##### Study method

As ventilator settings can affect aerosol drug delivery [7], the parameters set here were adjusted for their use in the ovine in vivo studies later. Ventilator parameters were fixed for all the in vitro experiments with a tidal volume of 500 ml, respiratory rate 14/min, square wave flow pattern, flow rate 40 lpm, positive end-expiratory pressure (PEEP) = 5 and FiO_2_ = 0.21.

**Fig. 1:**
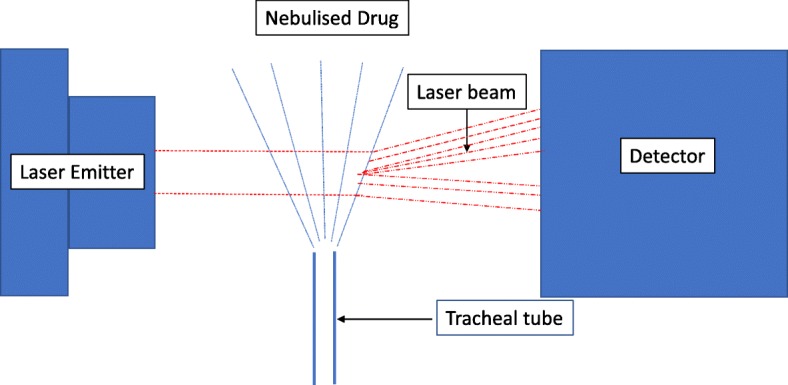
Principle of laser diffraction technique for particle sizing

**Fig. 2:**
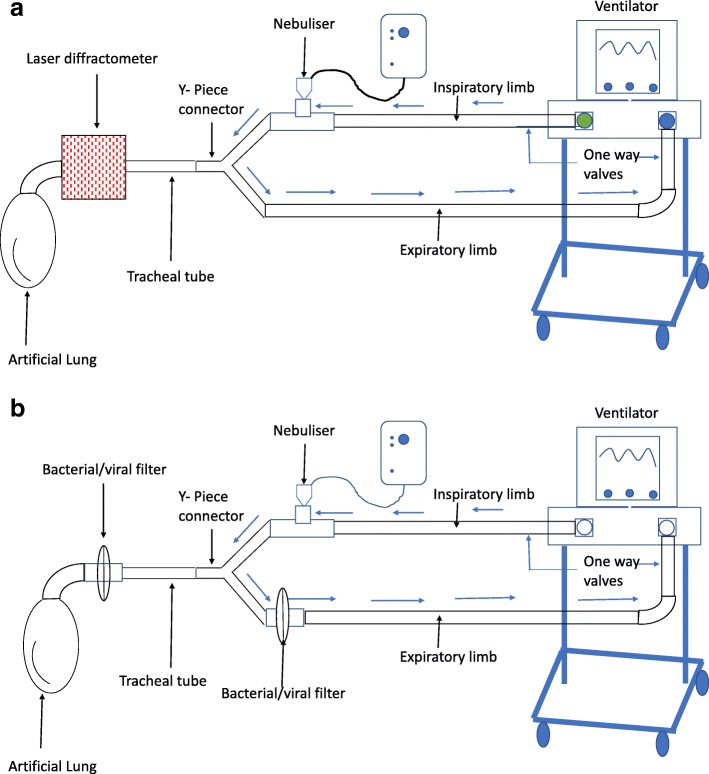
**a** Experimental set-up for particle sizing experiments. **b** Experimental set-up for inhaled mass experiments

As recommended by expert consensus, humidification was turned off for the experiments [21]. The tips of the tracheal tubes were placed in the receptacle of the laser diffractometer (Spraytec, Malvern Instruments, UK) using a silicone adaptor. The principle of the laser diffraction technique is shown in Fig. 1. The circuit was closed using an adult test lung (Dräger Medical, Lubeck, Germany) thus enabling continuous operation. The nebuliser was then filled with 4 ml tobramycin 100 mg/ml (400 mg) and then repeated with 4 ml of 40 mg/ml concentration (160 mg). Nebulisation was considered to be complete when no mist was visible from the nebuliser. The nebuliser and bacterial filter were changed after every experiment as per the guidelines [19]. Figure 2a demonstrates the study set-up in the laboratory.

##### Data collection and analysis

The following data were collected: baseline temperature, pH and viscosity of the tobramycin solution and nebulisation time. The viscosity of the tobramycin solution was determined at a constant temperature of 25 °C using a DHR-3 Rheometer (TA Instruments, USA). Particle size distributions were expressed as dv_10_ (volume diameter under which 10% of the sample resides), dv_50_ (volume median diameter) and dv_90_ (volume diameter under which 90% of the sample resides). In addition to dv_10_, dv_50_ and dv_90_, the percentages of particles between sizes 1 and 5 μm (the sizes conventionally considered for lung deposition) obtained from the laser diffractometer were included in this work.

#### In vitro nebulised mass study

##### Equipment

Adult-use mechanical ventilator, VMN, endotracheal tubes and tracheostomy tubes and tobramycin were used as per the particle size distribution study described above. In addition, respiratory (bacterial and viral) filters (filter pads, Fisher and Paykel, Auckland, New Zealand) were used.

##### Study method

Using a previously described methodology [22], we used the same set-up as described previously and insert the tip of the tracheal tube into a respiratory (bacterial and viral) filter (filter pads, Fisher and Paykel, Auckland, New Zealand). The circuit was closed using an adult test lung (Dräger Medical, Lubeck, Germany) thus allowing continuous operation. The nebuliser was filled with 4 ml tobramycin 100 mg/ml (400 mg) instilled with the nebulisation process to continue until there is no visible mist from the nebuliser. The ventilator was paused whilst the filter was removed from the circuit and stored at − 80 °C for drug assay using liquid chromatography with tandem mass spectrometry (LC/MS-MS). The process was repeated with 4 ml tobramycin 40 mg/ml (160 mg). The nebuliser was weighed before and after nebulisation to describe residual drug quantity. The experiment was done with each of the tracheal tubes as previously mentioned. Each experiment was repeated in triplicate. The nebuliser and bacterial filter were changed after every experiment as per the guidelines [19]. The study set-up was as shown in Fig. 2b.

##### Sample and data analysis

Tobramycin was extracted from the filter using water in a sonication bath, stored at − 80 °C and analysed later using liquid chromatography-tandem mass spectrometry (LC-MS/MS) as described later. The amount of drug was reported as milligrams (mg) of tobramycin. The percentage of the nebulised drug on the filter was calculated and expressed with ranges. Combined data from the particle sizing and nebulised mass study was used to describe the lung dose, i.e. the amount of drug in the effective particle size range for distal lung deposition.

### Development of an ovine model for aerosol studies

#### Design

We used a healthy ovine model to assess the regional drug deposition and lung ISF concentrations of intravenously and nebulised tobramycin.

#### Ethics

All the ovine studies have been ethically approved by the Queensland University of Technology animal ethics committee approval (Approval no. 1100000052; lung ISF study) and University of Queensland animal ethics committee approval (Approval no. SOM/268/16/RBWH; regional distribution studies).

#### Operating room set up

The basic operating room set-up and the anaesthesia and instrumentation components of the study were the same for the regional drug distribution study and the tobramycin concentration in the lung ISF study.

The operating room was equipped to manage and monitor for the animals’ health and well-being. A ‘head box’ or suspender was used to stabilise and immobilise the head whilst being sedated and mechanically ventilated in a prone position. Syringe drivers and infusion pumps were used for fluid infusions and anaesthesia maintenance. Precision microdialysis pumps (CMA 107, CMA microdialysis, Solna, Sweden) were used for microdialysis. A fibreoptic bronchoscope ((Pentax FB15TV, Philips, Tokyo, Japan) was used to obtain mini-bronchoalveolar lavage (BAL) samples. A qualified vet supervised the procedure at all times.

#### Anaesthesia and instrumentation

##### Pre-study

The veterinary specialist examined all Merino sheeps, and baseline blood samples were collected with only healthy Merino sheep weighing 40 to 50 kg selected for the study. The sheeps were fasted overnight and were led to the operating room in a protective sling with face, neck and chest area shaved.

##### Venous access, anaesthesia induction, airway access and mechanical ventilation

Using aseptic technique, a central venous line was inserted through the right jugular vein under local anaesthesia. Anaesthesia was commenced using induction agents, midazolam 0.5 mg/kg and alfaxalone 3 mg/kg (Jurox Pty Ltd., Hunter Valley, Australia). Orotracheal intubation was performed using an endotracheal tube (Portex, Smiths Medical, London, UK). Tracheal intubation was confirmed using a colorimeter and mechanical ventilation commenced. Ventilator parameters used throughout the experiment fulfilled the Acute Respiratory Distress Syndrome Network criteria for lung protective ventilation [23]. Initial ventilator settings for all the sheeps were respiratory rate 14 breaths per minute, tidal volume 500 ml, PEEP 5 cm H_2_O and fraction of inspired oxygen (FiO_2_) 0.21. During nebulisation, square wave flow pattern was applied, and the humidifier was turned off. Respiratory rate and FiO_2_ were adjusted according to arterial blood gas (ABG) results to maintain normal parameters. Due to the long length of the sheep airway, it was necessary to perform a tracheostomy to represent the human airway. This was performed with an aseptic technique using a size 9 tracheostomy tube (Portex, Smiths Medical, London, UK) and confirmed using bronchoscope and capnography. The airway was assessed using bronchoscopy for any anatomical variation or pathological abnormality. The animal was subsequently positioned in a prone position. Anaesthesia was maintained using ketamine 3–5 mg/kg/h, midazolam 0.25–0.5 mg/kg/h and alfaxalone 4–6 mg/kg/h infusion titrated to effect. Hydration was maintained using Hartmann’s solution at the rate of 2 ml/kg. The animal was kept warm using warming blankets. The operating room set-up, anaesthesia and instrumentation were common to the two ovine studies—regional drug distribution and microdialysis.

##### Monitoring

The sheep had routine monitoring using ECG, arterial blood pressure (ABP), end-tidal carbon dioxide monitor (ETCO_2_), pulse oximetry and central venous pressure (CVP) monitoring. An invasive arterial line was inserted in the facial artery using a cut-down method for the ABP monitoring and ABG sampling. Vital physiological data was recorded using Marquette Solar 8000 monitor (GE Healthcare, Little Chalfont, UK). A urinary catheter and orogastric tube were also inserted. Regular ABGs (two hourly) were obtained to confirm the adequacy of ventilation and other parameters such as electrolytes, glucose and lactate levels.

### Comparing regional pulmonary distribution of intravenous and nebulised tobramycin during mechanical ventilation

#### Computed tomography scan

Following adequate instrumentation, a CT scan of the chest was performed to confirm normal lung anatomy, and the images were used to obtain a region of interest (ROI) during image analysis. The ROI provides the lung outline for superimposition onto the gamma camera images for subsequent analysis.

#### Drug administration

##### Nebulisation

Using a disposable (VMN) (Aeroneb pro, Aerogen, Inc., Galway, Ireland) in the inspiratory limb of the ventilator circuit, tobramycin 400 mg (100 mg/ml) labelled with technetium-99m was nebulised until no further mist is visible. Previous studies have shown that the aerosol characteristics of labelled tobramycin are similar to that of unlabelled tobramycin [24].

##### Intravenous

Technetium-99m-labelled tobramycin 400 mg was injected via a central venous line as a 30-min infusion. The lumen was flushed with 100 ml of 0.9% saline.

##### Image acquisition

A single-head gamma camera (GE Starcam 600 XR/T Gamma Camera, GE Healthcare Global Diagnostic Imaging, Waukesha, WI, USA) was used to acquire planar images. The CT scan and the gamma camera scan equipment were located within the same research facility. Dynamic (10 s frames) and static scans were obtained. Images were acquired from lateral, dorsal and ventral aspects. Blood samples at predetermined intervals were obtained to coincide with the scans. Radiation count in the nebuliser and the syringe was performed before and after the nebulisation and intravenous administration, respectively, to define the dose and radioactivity administered.

##### Euthanasia and tissue sampling

At the completion of the study, the sheep was euthanized using sodium pentobarbitone (295 mg/ml, 0.5 ml/kg). Death was confirmed by loss of cardiac electrical activity and ABP trace. After euthanasia, the organs were surgically retrieved. Direct tissue sampling from pre-defined regions of the lungs, liver, both kidneys and urine was performed. The remains of the animals were frozen and stored until disposed of via incineration.

##### Data analysis

The images were analysed and reported as radioactivity distribution into peripheral to central ratio (P/C ratio), also known as ‘penetration index’ for the nebulised tobramycin group. For this analysis, the central region represents the conducting airways, and the peripheral represents the alveoli. Parameters used to compare the intravenous and nebulised route of administration include dorsal to ventral ratio, right to left lung ratio and upper/middle/lower lung zone distribution. The quantitative analysis included describing the radioactivity in per gram of pulmonary and extra-pulmonary tissue. Blood radioactivity was compared between the two groups as a surrogate measure of drug level in the systemic circulation.

### Calibration of the microdialysis system using in vitro microdialysis recovery study

#### Equipment

The study used fresh frozen plasma and a magnetic stirrer CMA 63 microdialysis catheters (molecular weight cut-off of 20 kDa; an outer diameter of 0.6 mm and a membrane length of 30 mm; CMA Microdialysis AB, Stockholm, Sweden), CMA 107 precision microinfusion pump (CMA Microdialysis AB, Stockholm, Sweden) and Cole-Parmer two-syringe infusion pump 230 VAC CE (John Morris Group, Chatswood, Australia).

#### Study method

Microdialysis probes were perfused with Ringer’s solution at three different flow rates 1, 1.5 and 2 μl/min using the precision pump. The membranes of the microdialysis probes were immersed in 50 ml solution of 5 μg/mL of tobramycin and 5 μg/mL vancomycin concentrations. The samples were collected at 20-min intervals for 100 min with experiments repeated in triplicate.

#### Sample analysis

Samples were stored at − 80 °C and assayed using LC-MS/MS methods described previously.

Recovery rates were expressed as relative recovery (RR):

$$ \mathrm{RR}\ \left(\%\right)=\left(1-{C}_{\mathrm{dialysate}}/{C}_{\mathrm{solution}}\right)\times 100 $$

where *C*_dialysate_ = concentration of antibiotic in the microdialysate and *C*_solution_ = concentration of antibiotic in the test solution.

**Fig. 3:**
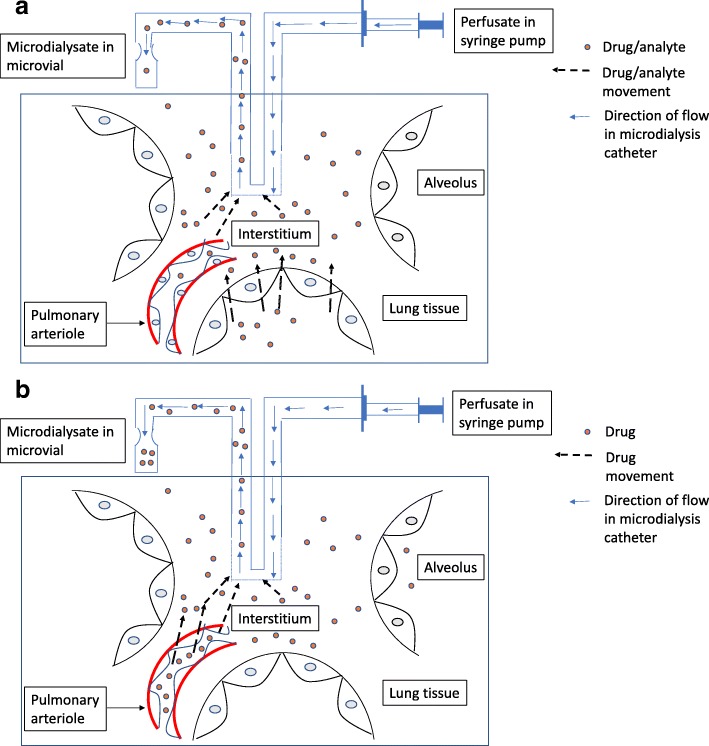
Schematic diagram demonstrating the principle of lung microdialysis. The unidirectional perfusate flow provides a concentration gradient for diffusion of analytes across a semi-permeable membrane placed in the lung interstitium. The dialysate is then collected in microvials for analysis. **a** Principles of lung microdialysis with nebulised antibiotics—showing higher antibiotic concentration in the alveoli. **b** Principles of lung microdialysis with intravenous antibiotics—showing higher antibiotic concentration in the pulmonary arteriole

Figure 3a, b describes the principles of microdialysis when used for the study of inhaled antibiotics and intravenous antibiotics, respectively. The principles have been described in detail previously [25].

### Comparing lung ISF concentrations of intravenous and nebulised tobramycin

#### Microdialysis catheter priming and set-up

Various factors influence in vivo recovery of analytes; hence, in addition to in vitro studies, it is essential to perform in vivo recovery [25]. Methods for in vivo recovery calculations have been described in the literature [26]. Internal indicator technique was used in this study. The perfusate for microdialysis was prepared using 0.1 ml (4 mg) of gentamicin 80 mg/2 ml vial. This was injected into a 1-l bag of Hartmann’s solution to make up a concentration of 4 μg/ml which was then be used as the perfusate for the catheters. Gentamicin was used as an internal standard to enable in vivo recovery calculations and was chosen due to similarities with tobramycin (molecular weight of gentamicin = 467 vs tobramycin = 477 g/mol). Using CMA 107 battery pumps, the microdialysis catheters were primed with the perfusate solution at a rate of 5 μl/min for 10 min. The catheters were then inserted, and the perfusate rate was reduced to 1 μl/min for the remainder of the experiment. Once inserted, the catheters were perfused for a period of 1 h to achieve a steady state equilibrium.

#### Insertion of microdialysis catheters

After anaesthesia induction and instrumentation, bilateral thoracotomies were performed in the fifth intercostal space exposing the interlobar fissures. A CMA 63 microdialysis catheter (CMA Microdialysis AB, Stockholm, Sweden) with a molecular weight cut-off of 20 kDa, an outer diameter of 0.6 mm and a membrane length of 30 mm was used. Four microdialysis catheters were inserted, in the right upper and lower and the left upper and lower lobes using an introducer needle under direct vision. Bioglue (Coseal surgical sealant, Cohesion technologies, US) was applied at the lung puncture site to minimise catheter movement post-insertion. An intercostal catheter was inserted in each of the pleural spaces and low-pressure suction applied, and the thoracotomy incisions will be closed. An intravascular microdialysis catheter, CMA 64 (CMA Microdialysis AB, Stockholm, Sweden) with a molecular weight cut-off 20 kDa and a membrane length of 20 mm, was inserted in the left jugular vein through a percutaneously inserted 18G cannula (B. Braun Australia Pty Ltd., Bella Vista, Australia).

#### Drug administration

##### Nebulisation

Using a disposable (VMN) (Aeroneb pro, Aerogen, Inc., Galway, Ireland) in the inspiratory limb of the ventilator circuit, tobramycin 400 mg (Tobra-day, Phebra 500 mg/5 ml) was nebulised. The nebulisation was continued until the visible mist from the nebuliser stopped. A baseline pre-nebulisation microdialysis sample was collected (T0).

##### Intravenous antibiotic administration

Tobramycin 400 mg (Tobra-day, Phebra 500 mg/5 ml) was administered intravenously through the central venous line as a 30-min infusion. A baseline pre-intravenous administration, microdialysis sample was collected (T0).

Following the drug administration (nebulised and intravenous), the samples were collected at every 20 min in a microvial (CMA, Microdialysis AB) for 8 h. The samples were stored at − 80 °C for further analysis.

##### Bronchoalveolar lavage

A mini-BAL was performed at *T* = 60 min, 4 h and 7 h using two 20 ml aliquots of normal saline as previously described [27, 28]. This procedure was performed using a fibreoptic bronchoscope with the tip wedged in the right lower lobe and left lower lobe bronchi. The actual epithelial lining fluid (ELF) concentrations of tobramycin were obtained from the measured BAL fluid concentrations after correction for dilution according to the equation:

$$ {C}_{\mathrm{ELF}}={C}_{\mathrm{BAL}}\ \left({\mathrm{Urea}}_{\mathrm{PLASMA}}/{\mathrm{Urea}}_{\mathrm{BAL}}\right) $$

where *C*_BAL_ corresponds to the tobramycin concentrations measured in the BAL fluid, and Urea_BAL_ and Urea_PLASMA_ correspond to the concentrations of urea determined in BAL fluid and plasma, respectively. Blood samples were taken for measuring urea to assess the adequacy of BAL.

##### Euthanasia, catheter inspection and organ retrieval

At the completion of the study, the sheeps were humanely euthanized using sodium pentobarbitone (295 mg/ml, dose 0.5 ml/kg). Using chest retractors, the microdialysis catheters were exposed and inspected to confirm that the catheters remained inside the lungs throughout the procedure. The catheters were inspected for obvious evidence of damage. The lungs were retrieved, and the sections of bilateral upper and lower lobes were visually inspected and harvested for further drug concentration analysis. The remains of the animals were frozen and stored until disposal via incineration.

##### LC-MS/MS method for sample analysis

The tobramycin concentrations in microdialysis samples reported were measured by an LC-MS/MS method using a simple dilution preparation and HILIC chromatography on a Shimadzu Nexera2 system coupled to a Shimadzu 8030+ triple quadrupole mass spectrometer (Kyoto, Japan). The assay method has validation for linearity (quadratic line from 0.1 to 20 μg/ml with precision and accuracy of all calibration standards within 8%), LLOQ (precision and accuracy within 6% at 0.1 μg/mL), matrix effects (normalised matrix factor precision within 9% at 0.2 and 16 μg/ml) and precision and accuracy (precision and accuracy within 10% at 0.3, 2 and 16 μg/ml) using the FDA criteria for bioanalysis [29]. Recovery data was used to calculate the corrected tobramycin concentrations in the microdialysate.

##### Statistical considerations

The results were expressed as mean, median, minimum, maximum and standard deviation. For the in vitro particle size and inhaled mass study, multiple linear regression was used to estimate the effect of the variables on the outcomes. For the in vitro microdialysis recovery studies, a linear regression model using recovery as the dependent variable and flow rate as the independent variable was applied. Similarly, the blood radioactivity levels in the ovine study were compared using Bayesian regression analysis using a random intercept for each sheep and Bayesian *p* values were obtained. These analyses were conducted using R version 3.4.2 and WinBUGS version 1.4.3 for the Bayesian regression analysis. The individual studies were not powered to detect statistical significance, and instead, we used confidence intervals where appropriate and discuss the size of the differences in terms of their clinical as well as statistical significance. The plots of summary statistics were used to compare groups and results over time.

## Results

The simulated adult mechanical ventilation model to evaluate the particle size distribution and quantify the efficacy of the aerosol system showed that fine particle fraction (1 to 5 μm) was significantly higher for 100 mg/ml solution compared to 40 mg/ml solution (99.4 ± 0.3% vs 82.3 ± 6%; *p* < 0.001). The lung dose delivered was significantly higher for 100 mg/ml solution compared to 40 mg/ml solution (132.7 ± 12.6 mg vs 39.2 ± 1.1 mg; *p* < 0.001). Nebulisation duration was significantly higher for the 100 mg/ml solution compared to 40 mg/ml solution for endotracheal tube (18.56 ± 2.36 vs 7.88 ± 1.08 min; *p* < 0.001) and tracheostomy tube (14.63 ± 1.14 vs 12 ± 1.4 min; *p* < 0.001). Tracheal tube type had a minimal effect which was not statistically significant, on the aerosol delivery parameters. Other parameters like dv_10_ and dv_95_ were not statistically different. Table 2 summarises the differences between the two formulations. The experiments were performed at a temperature of 25 °C.

**Table 2:** Comparative characteristics between tobramycin 40 and 100 mg/ml solutions

Parameters	Tobramycin 40 mg/ml	Tobramycin 100 mg/ml
pH 88	6.88	6.99
Viscosity (centipoise)	1.58	2.12
Duration of nebulisation (min)		
Endotracheal tube	7.88 ± 1.08	18.56 ± 2.36*
Tracheostomy tube	12 ± 1.4	14.63 ± 1.14*
Dv_10_ (μm)	1.0 ± 0.05	1.4 ± 0.04
VMD (μm)	1.8 ± 0.2	2.1 ± 0.08
Dv_95_ (μm)	5.7 ± 0.2	3.6 ± 0.8
FPF (%)	82.3 ± 6	99.44 ± 0.3*
Lung dose (mg)	39.2 ± 1.1	132.7 ± 12.6*

*dv*_*10*_ volume diameter under which 10% of the sample resides; *VMD* volume median diameter under which 50% of the particles resides; *dv*_*95*_ volume diameter under which 95% of the sample resides; *FPF* fine particle fraction (1–5 μm)

*p < 0.05

**Fig. 4:**
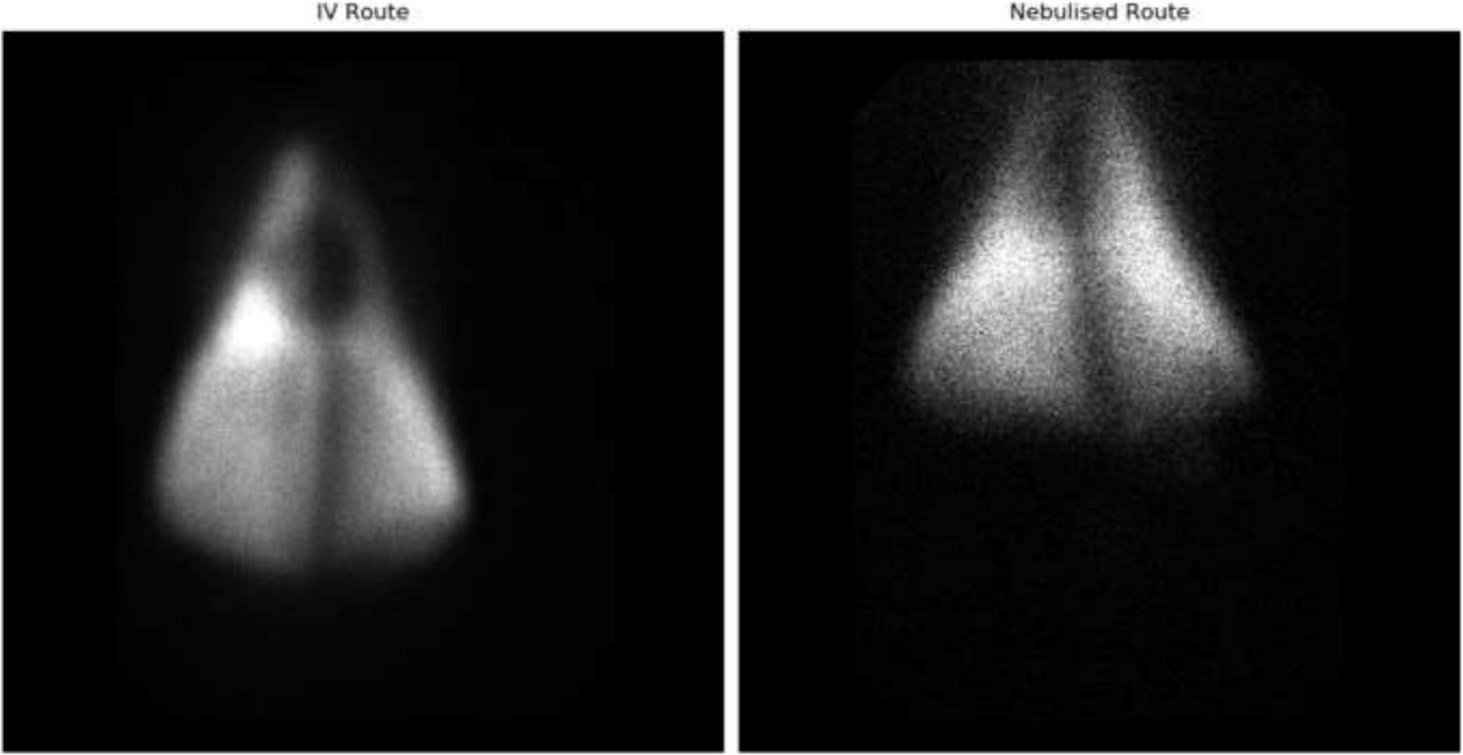
Comparative dorsal scintigraphy images of lung deposition of i.v and nebulised radiolabelled tobramycin

The ovine model for the comparative regional lung distribution study between aerosolized and intravenous tobramycin was successfully established. Female sheeps (ewes, *n* = 4) were used for the study. There were no adverse events documented during the ovine studies. For the nebulised sheep, the P/C ratio (penetration index) was 0.9 for the right lung and 0.87 for the left lung. The blood radioactivity was significantly higher for the sheep with intravenous tobramycin compared to that for the nebulised tobramycin (209.2 ± 97.6 Mbq/ml vs 33.3 ± 5.4 Mbq/ml; *p* < 0.001). The right to left ratio was 0.9 for intravenous and 0.89 for the nebulised sheep. Figure 4 shows the comparative gamma camera scan images between i.v. and nebulised technetium-labelled tobramycin. Table 3 shows that the liver, kidney and urine levels were lower for nebulised compared to intravenous sheep.

**Table 3:** Quantitative extra-pulmonary tissue radioactivity concentrations

Organ	Nebulised tobramycin	Intravenous tobramycin
Liver (Bq/g/Mbq)	76.5	2541.7
Left kidney (Bq/g/Mbq)	585.8	1048.4
Right kidney (Bq/g/Mbq)	704.2	2126.7
Urine (Bq/g/Mbq)	1479.1	7866.3

*Bq/g/Mbq* becquerel per gram per megabecquerel of administered dose

Microdialysis recovery in the simulated in vivo conditions showed that relative recovery for vancomycin was 32.6 ± 0.09%, 27.5 ± 0.09% and 20.7 ± 0.04% for perfusate flow rates of 1, 1.5 and 2 μl/min, respectively. Thus, for vancomycin, the highest relative recovery was for a perfusate flow rate of 1 μl/min (95% CI 24.8 to 35.7%). For tobramycin, the recoveries were 69 ± 3.74%, 69.5 ± 3.47% and 65 ± 3.8%, respectively. The differences were not seen to be statistically significant.

**Fig. 5:**
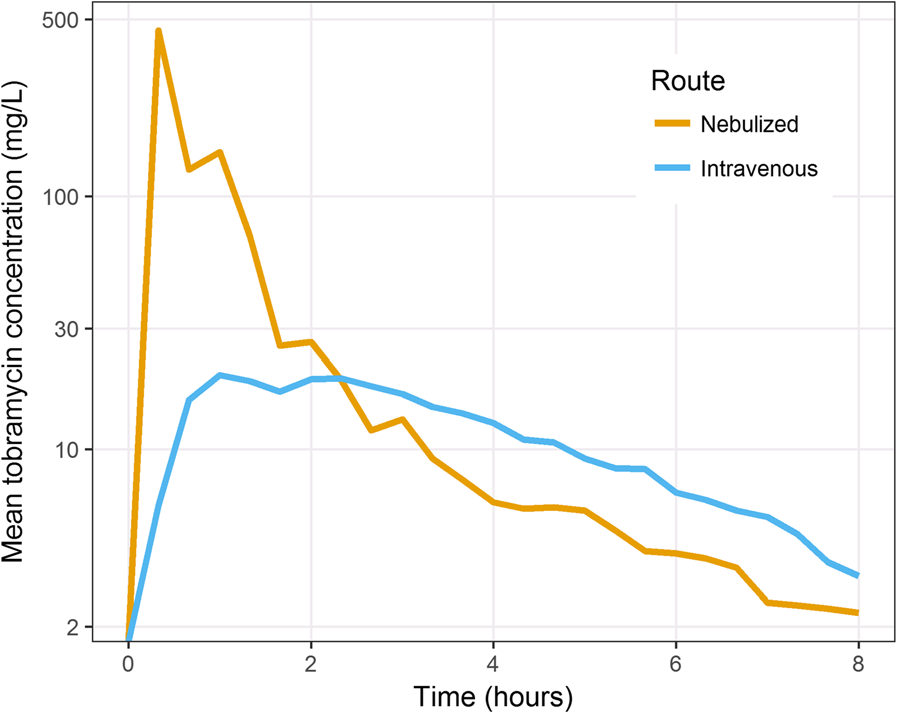
Comparative lung concentrations of tobramycin between nebulised and intravenous route of administration

The lung isf concentrations in the mechanically ventilated ovine model showed that area under the concentration- time curve (mean) and peak (or maximum) concentration in the concentration- time curve (mean) for nebulized tobramycin was 313 mg-h/liter and 535.8 mg/liter and for intravenous tobramycin the values were 90.82 mg-h/liter and 22.2 mg/liter respectively, for intravenous tobramycin. Figure 5 shows lung ISF concentrations with nebulised tobramycin compared to intravenous tobramycin. For the nebulised tobramycin sheep, ELF concentration was 1.94 mg/l at 1 h and for intravenous tobramycin sheep, and the level was 0.19 mg/l. (Table 4).

**Table 4:** Comparative epithelial lining fluid concentrations for nebulised and tobramycin sheep at two time points, 1 and 6 h post-antibiotic administration

ELF time points	Nebulised tobramycin	Intravenous tobramycin
1 h (mg/l)	1.94	0.19
6 h (mg/l)	0.07	0.38
AUC_ELF_ (mg-h/l)	5.99	1.52
AUC_plasma_ (mg-h/l)	56.88	53.60
AUC_lung_/AUC_plasma_ (mg-h/l)	0.11	0.03

*ELF* epithelial lining fluid; *AUC* area under the concentration-time curve

Technically the ovine model set up for the regional lung distribution and lung microdialysis study was uneventful. There were no adverse events affecting the interpretation of results. We have optimised anaesthesia, ventilation, surgical instrumentation, scintigraphy protocols, sample collection and analyses in this model.

## Discussion

This paper describes an incremental model of the study of the PK of nebulised antibiotics. As the lung is a heterogeneous organ, PK studies to accurately characterise drug concentrations in the various compartments are difficult. We have used a methodological approach for the assessment of the PK of nebulised antibiotics using in vitro and ovine models. For nebulised antibiotic administration, important PK parameters are pulmonary deposition efficiency, regional distribution, pulmonary absorption and residence time [6]. For therapeutic response, the dose deposited at the site of infection in the lung is important [30] particularly the unbound concentration of the drug [6]. We studied these essential aspects of nebulised antibiotic therapy using tobramycin.

For higher concentration tobramycin (100 mg/ml), the aerosol delivery system can deliver a higher lung dose. This is important for tobramycin and other aminoglycosides that rely on peak (or maximum) concentration as the PK measure for drug effect. Aerosol particle size and the aerosolized drug mass are important parameters for assessing the efficacy of an aerosol delivery system [7]. Methods for the study of the aerosol particle size distribution and nebulised mass have been summarised previously [22, 31]. We used the laser diffractometer which is a validated technique for the assessment of aerosol particle size distribution [32] and an established technique for the nebulised mass study [22]. Recent recommendations have been to place the VMN 10–15 cm from the Y-piece [33]. The VMN was placed just before the Y-piece in the inspiratory limb to mimic local and international practices [34] as well as the manufacturer’s recommendations.

Our results suggest that mechanically ventilated sheep could be effectively used for the study of regional lung distribution studies especially for drugs administered for local effect on the lung diseases, e.g. infections. The distribution parameters of nebulised tobramycin would support its use in lung infections especially with minimal systemic and extra-pulmonary tissue levels as seen in our study. Methods of assessing pulmonary drug distribution have been summarised in detail previously [35]. Whilst single-photon emission computed tomography (SPECT) and positron emission tomography (PET) provide a 3D description of the regional drug distribution in the lungs, for most purposes, planar gamma scintigraphy is sufficient [36, 37] and feasible; therefore, we used the gamma scintigraphy method for our study.

The results of the present microdialysis calibration study suggest that large molecules like vancomycin have significantly reduced recovery and protein binding adversely affected the recovery. Perfusate flow rates also affected relative recovery rates for tobramycin and vancomycin. Thus, microdialysis-based studies for large molecules as well as highly protein-bound substances should be evaluated in vitro in a similar model. By calibrating the microdialysis system, thus depending on the study drug, it will enable the correct combination of microdialysis catheters and perfusate flow rates. The techniques used in other studies to assess pulmonary drug concentrations used could not inform adequately the longitudinal data desired over time. These studies have been previously summarised [25], but most studies employed antibiotic BAL concentrations as a surrogate [38, 39]. Some authors have used blood concentrations to perform population PK compartmental modelling [40]. For most bacterial infections, the antibiotic site of action is the ISF [41]. Lung microdialysis is an established technique to sample lung ISF for the purposes of measuring antibiotic concentrations [25]. The details of the microdialysis technique and relevant considerations have been described elsewhere [42–45]. Recovery studies are important for the accurate calculation of the antibiotic concentration in the microdialysis sample [26].

For tobramycin, the high lung ISF concentration achieved with nebulised route meets the PK requirements for drug effect. Study of drugs with different PK parameters would elucidate if the nebulised route is appropriate for therapy. Preclinical models for the study of pulmonary drug delivery have been well described [10]. Methods such as in vitro cell culture whilst providing a consistent method of assessing nebulised drugs cannot predict the pulmonary bioavailability of the drug as it cannot assess the other barriers to absorption [10]. In vitro cell culture method remains a viable alternative to test for drug toxicity. Isolated perfused lung model provides an ex vivo model for drug PK assessment but lack of tracheobronchial circulation could be a drawback in the assessment of systemic absorption [10]. In vivo models provide the most thorough quantitative and qualitative data to inform the PK of pulmonary drug delivery [46].

As airway size and type were considered an important variable in drug deposition, the in vitro aerosol characteristics were investigated with the size 9.0 mm I.D. tracheal tubes as these were the tracheal tube sizes used for the in vivo studies in sheep, thus minimising the variables in the study data.

Sheep are the ideal large animal model to study the PKs of nebulised antibiotics [12]. Their size and lung anatomical-physiological features are similar to humans [47]. By replicating the study, data could be acquired to describe the PK profile of a wide range of nebulised antibiotics. This adds valuable information for potential efficacy and toxicity of drugs and formulations. Our model can be applied to other potentially important questions and test other factors such as variations in ventilator parameters, the distribution characteristics of newer nebulised drug formulations, novel delivery devices and different patient parameters such as posture that might affect pulmonary drug delivery. There is potential for the study of other nebulised drugs where lung ISF concentrations are important, e.g. other anti-infectives like antiviral and antifungal agents, chemotherapeutic agents and immunomodulatory drugs.

Future work will consist of pharmacodynamic studies in a variety of clinically important experimental pneumonia settings. This data could be used to improve the precision and accuracy of nebulised antibiotic dosing schedules in a variety of lung infections in the pre-clinical models. The complexity of the pathophysiological changes associated with pneumonia makes the development and management of the animal models of experimental pneumonia challenging. Factors that need consideration include ethics, selection of appropriate animal species, number of subjects, inoculation routes and sampling issues, and the present animal model used account for some, but not all, of these. Clinical trials using accurate information from these mechanistic and preclinical studies could yield better therapeutic outcomes.

## Conclusions

This program of research aims to apply in vitro and ovine models for the study of nebulised antibiotic delivery and lung PK. This model can be used for further studies involving drugs where knowledge of pulmonary drug concentration is essential. Systematic investigation of other aspects of nebulised antibiotic therapy using this model will introduce further refinements in nebulised antibiotic therapy thus improving patient care and outcomes.

## Abbreviations

ABG: Arterial blood gas
ABP: Arterial blood pressure
BAL: Bronchoalveolar lavage
C/P: Central to peripheral ratio
CT: Computed tomography
CVP: Central venous pressure
ELF: Epithelial lining fluid
ETCO_2_: End-tidal carbon dioxide
ISF: Interstitial fluid
LC-MS/MS: Liquid chromatography-tandem mass spectrometry
PD: Pharmacodynamics
PEEP: Positive end-expiratory pressure
PET: Positron emission tomography
PK: Pharmacokinetics
ROI: Region of interest
RR: Relative recovery
SPECT: Single-photon emission computed tomography
VAP: Ventilator-associated pneumonia
VMN: Vibrating mesh nebuliser
